# Supplementary material for: Gut Microbiota and Lipid Metabolism in Bullfrog Tadpoles: A Comparative Study Across Nutritional Stages
Source: Microorganisms. 2025 May 15;13(5):1132. doi: 10.3390/microorganisms13051132 (PMC12113880; doi:10.3390/microorganisms13051132)
Supplement: Supplementary file 1 [file microorganisms-13-01132-s001.zip › Table S1.pdf]

Table S1. Primers and sequences mentioned in this experiment

Genes	Primer sequence (5′-3′)	GenBank reference	PL (bp)
PPAR-γ	F: AGGTCCCCTTCCCTTCAAGA	XM_040359320.1	110
	R: CAACCTGGTCCAACACGGTA		
PPAR-α	F: CGTCCTGGACTGCTCAATGT	XM_040345778.1	141
	R: CCTCAGGTCGGCCATTTTCT		
FAS	F: AAGCCGTGAGAAGTGTCTGG	XM_040330786.1	136
	R: CTGACGCCGCTTTTCACAAA		
DGAT1	F: TCCAGAACTCCATGAAGCCG	XM_040334894.1	166
	R: TCCCGATCTCCAAAACGCAT		
HSL	F: TACTAACAGAACGCCAGGGGA	XM_040327281.1	149
	R: GCCCACCTCAGCTTTAGACTC		
CPT1	F: CCCAGATGCCTTTGTCCAGT	XM_040328372.1	83
	R: TGGAGGCCTCATACGTCAGA		
ACOX1	F: GCGATGACGCACAGTGTAAG	XM_040329976.1	129
	R: GGTCATAGGTGGCAGTCGTC		
ACTB	F: AAGCTCAAAGCAAGAGGGGT	XM_040357114.1	132
	R: GTTCCTCTGGGGCAACTCTC		

Note: *PPAR-γ*: peroxisome proliferators activated receptor-γ, *PPAR-α*: peroxisome proliferators activated receptor-α, *FAS*: fatty acid synthase, *DGAT1*: diacylglycerol O-acyltransferase 1, *HSL*: hormone-sensitive lipase, *CPT1*: carnitine O-palmitoyltransferase-1, *ACOX1*: acyl-CoA oxidase, PL: product length.
