# Supplementary material for: Gut Microbiota and Lipid Metabolism in Bullfrog Tadpoles: A Comparative Study Across Nutritional Stages
Source: Microorganisms. 2025 May 15;13(5):1132. doi: 10.3390/microorganisms13051132 (PMC12113880; doi:10.3390/microorganisms13051132)
Supplement: Supplementary file 1 [file microorganisms-13-01132-s001.zip › Table S2.pdf]

Table S2. One-way ANOVA test statistic values for biochemical and enzymatic indicators and mRNA expression levels of lipid metabolism-related genes in bullfrog tadpoles.

Items	0d vs 10d	0d vs 20d	10d vs 20d
ALT	0.868	0.036	0.036
AST	0.628	0.018	0.035
TG	<0.001	<0.001	0.012
TC	0.141	<0.001	0.001
NEFA	<0.001	0.042	<0.001
HDL-C	0.203	<0.001	<0.001
LDL-C	0.288	<0.001	<0.001
fas	0.597	0.001	0.003
dgat1	0.885	0.011	0.019
ppar-α	0.244	0.029	0.211
acox1	0.399	0.181	0.539
hsl	0.923	0.010	0.026
cpt1	0.399	0.030	0.080
ppar-γ	0.896	0.011	0.010
