# Supplementary material for: Gut Microbiota and Lipid Metabolism in Bullfrog Tadpoles: A Comparative Study Across Nutritional Stages
Source: Microorganisms. 2025 May 15;13(5):1132. doi: 10.3390/microorganisms13051132 (PMC12113880; doi:10.3390/microorganisms13051132)
Supplement: Supplementary file 1 [file microorganisms-13-01132-s001.zip › Table S3.pdf]

Table S3. One-way ANOVA test statistic values for α-diversity indices of bacterial communities across different habitats at the operational taxonomic unit level.

Items	Habitats	0d vs 10d	0d vs 20d	10d vs 20d
Shannon	Gut	0.025	0.746	0.113
	Water	0.416	0.001	0.012
	Excrement	0.995	0.059	0.047
Simpson	Gut	0.124	0.438	0.422
	Water	0.724	<0.001	<0.001
	Excrement	0.259	0.445	0.061
Chao1	Gut	0.009	0.641	0.074
	Water	0.001	<0.001	0.701
	Excrement	<0.001	<0.001	0.847
Pielou_e	Gut	0.051	0.888	0.121
	Water	0.754	<0.001	0.002
	Excrement	0.622	0.193	0.025
Goods_coverage	Gut	0.214	0.878	0.026
	Water	0.001	<0.001	0.464
	Excrement	<0.001	<0.001	0.809
