# Supplementary material for: Gut Microbiota and Lipid Metabolism in Bullfrog Tadpoles: A Comparative Study Across Nutritional Stages
Source: Microorganisms. 2025 May 15;13(5):1132. doi: 10.3390/microorganisms13051132 (PMC12113880; doi:10.3390/microorganisms13051132)
Supplement: Supplementary file 1 [file microorganisms-13-01132-s001.zip › Table S4.pdf]

Table S4. Adonis analysis between groups

VS Group	F.Modle	R2	Pr(>F)
G-E vs G-M	27.6823	0.6804	0.002
G-E vs G-X	31.9493	0.7107	0.002
G-M vs G-X	11.5405	0.4518	0.001
E-E vs E-M	11.7449	0.4562	0.001
E-E vs E-X	44.0346	0.7587	0.001
E-M vs E-X	9.09547	0.3938	0.001
W-E vs W-M	113.171	0.9339	0.008
W-E vs W-X	226.126	0.9658	0.008
W-M vs W-X	72.8608	0.8793	0.003

Note: F. Model represents the F-test value; R2 represents the explanatory power of different groups on sample differences, that is, the ratio of group variance to total variance. A larger R2 indicates a higher explanatory power of the group on differences; Pr represents the *P*-value, which is less than 0.05, indicating a high reliability of this test.
