# Supplementary figures and images for: Gut Microbiota and Lipid Metabolism in Bullfrog Tadpoles: A Comparative Study Across Nutritional Stages
Source: Microorganisms. 2025 May 15;13(5):1132. doi: 10.3390/microorganisms13051132 (PMC12113880; doi:10.3390/microorganisms13051132)

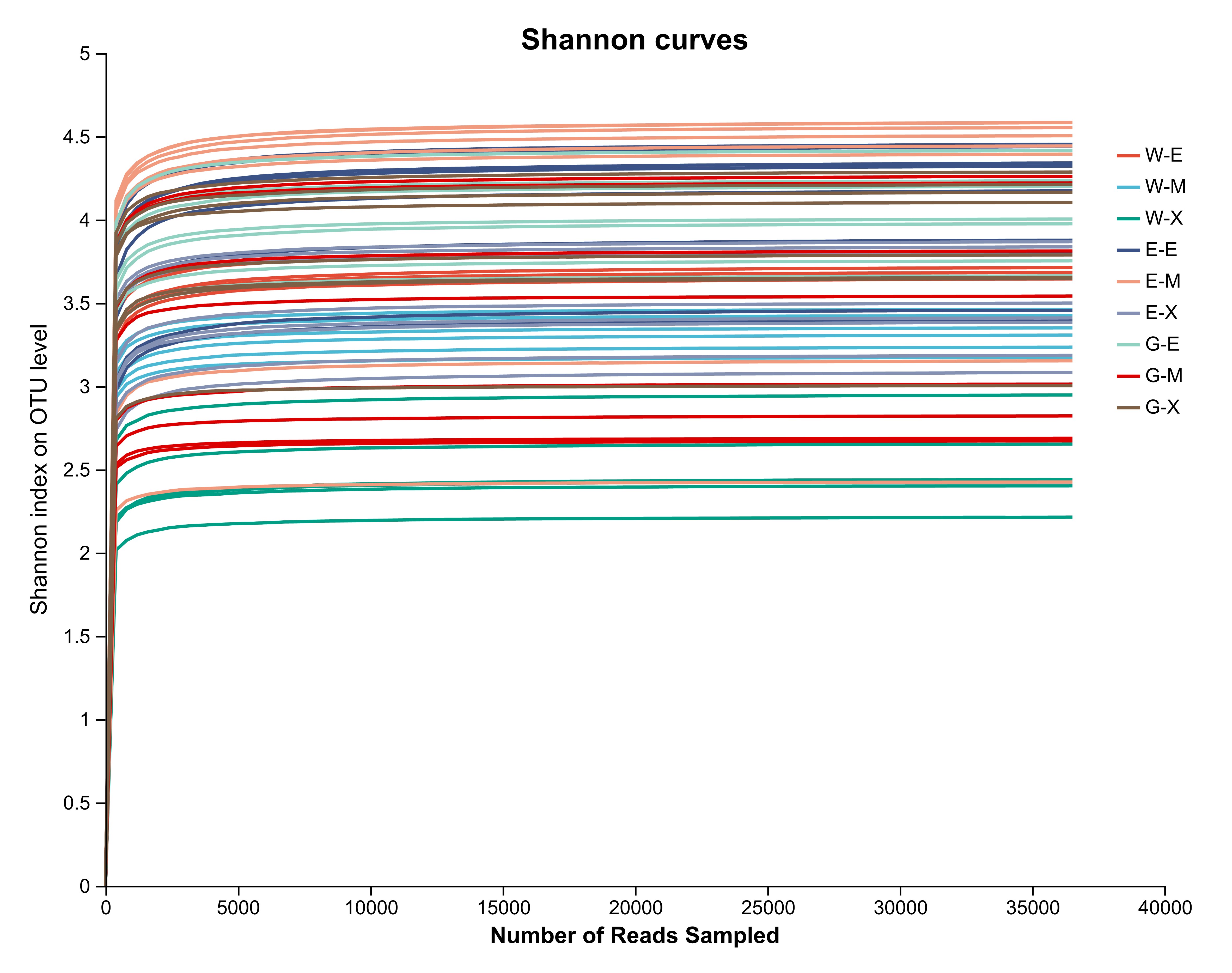

Supplement: Supplementary file 1 [file microorganisms-13-01132-s001.zip › Figure S1.jpg]
